# Analysis of Dispensing Errors Made by First-Year Pharmacy Students in a Virtual Dispensing Assessment

**DOI:** 10.3390/pharmacy9010065

**Published:** 2021-03-20

**Authors:** Sara Chuang, Kate Lorraine Grieve, Vivienne Mak

**Affiliations:** Faculty of Pharmacy and Pharmaceutical Science, Monash University, Parkville, VIC 3052, Australia; Sara.Chuang@monash.edu (S.C.); kgri0002@student.monash.edu (K.L.G.)

**Keywords:** dispensing, harm, MyDispense, education, simulated learning environment

## Abstract

Pharmacists have a crucial role in the supply of medications and ensuring optimal patient outcomes. However, with the increased use of prescription medications, there is a potential for dispensing errors to occur. Some dispensing errors can result in patient harm, with some leading to death. The development of safe and accurate dispensing skills in pharmacy students is an essential part of the pharmacy curriculum to prevent such dispensing errors from occurring. A retrospective study was conducted on a virtual dispensing assessment completed by first-year pharmacy students using MyDispense at Monash University. Students were assessed on their ability to safely and accurately dispense four prescriptions. The students’ answers in the assessment were then analyzed using qualitative and quantitative methods. Errors in drug quantity, number of repeats, product, patient and prescriber selection were quantitatively analyzed. Through the development of a codebook, frequency of errors was determined for label directions and appropriate use of ancillary labels. In this study, the dispensing errors that were identified depended on the class of medication. Errors in label directions were most common, with the majority of errors displaying incorrect route of administration, drug formulation and/or frequency of dosing. Identified errors were then further categorized into potential severity of harm, ranging from “no harm” to “severe harm”. The findings from this study show the types of errors made by students that are preventable and the potential for first-year pharmacy students to benefit from more comprehensive introductions to dispensing guides and safe environments to practice.

## 1. Introduction

Pharmacists are medicine experts and have a crucial role in the safe supply of medications via dispensing. The process of dispensing medicines involves reviewing a prescription for appropriateness, establishing clear label instructions to reflect the prescriber’s intentions and verifying that the medication is given to the correct patient [1]. Medication errors may arise during the dispensing process and are defined as “any preventable event that may cause or lead to inappropriate medication use or patient harm” [2]. Australia had a total of 208.5 million government subsided prescriptions dispensed between 2019 and 2020 [3].With such a high volume of prescriptions dispensed annually, the potential for medication errors to occur increases. 

In a review conducted by James et al. [4], the most common dispensing errors identified in community and hospital pharmacies were incorrect medication, strength, form or quantity or incorrect directions on the medication label. This was further demonstrated by studies within community settings where common dispensing errors not only included the previously mentioned errors but also incorrect patient selection [5]. Dispensing errors can cause a range of undesirable patient outcomes including, but not limited to, adverse drug reactions, drug–drug interactions, failure of treatment or, in severe cases, mortality [6]. While not all medication errors result in harm, this can lead to patient dissatisfaction and loss of trust in the healthcare system [7]. This highlights the importance of accurate and safe dispensing practice in pharmacy.

As such, teaching appropriate dispensing practices is essential in the development of dispensing skills in pharmacy students. The use of simulation is one of many ways to teach dispensing skills. MyDispense is a virtual platform developed by Monash University to provide a safe learning and teaching environment for dispensing and has been widely distributed and implemented globally [8]. Utilizing a simulated environment, students are given an authentic dispensing experience, contextualizing the theory learnt as part of their curriculum [9]. The use of a simulated environment allow students to better understand and identify medication and prescription errors [10], without the “real-life” consequences of dispensing errors. A study showed students found MyDispense to be an effective tool for learning prior to commencement of placements [11] and assessments [12].

MyDispense is used in the second semester of the 1st year of the pharmacy degree at Monash University, where medication dispensing is first taught. Prior to this, students cover the fundamentals of pharmaceutical chemistry, biology, physiology and pharmacy practice. The aims of this study were to identify the most common dispensing errors made by first-year pharmacy students and to identify how these errors may impact patient outcomes if done in practice. To further explore the extent of these dispensing errors, the Harm Associated with Medication Error Classification (HAMEC) tool developed by Gates et al. [13] was used to categorize the errors and the potential impact on patient safety. The findings from this study therefore aim to inform future research into potential strategies or curriculum changes that may be required to improve safe dispensing skills in pharmacy students.

## 2. Materials and Methods

### 2.1. Research Participants

Participants for this study were first-year pharmacy students at Monash University in Melbourne, Australia, who completed the MyDispense assessment. Data from three pharmacy student cohorts from 2017 to 2019 were included in this study. Students complete 3 weeks of Safe Medication Practice topics prior to the assessment. These topics begin from Week 5 of the second semester of the 1st year. These learning materials in these topics cover dispensing practices, and each week consists of 2 h of pre-class learning, two 1 h lectures and one 2 h workshop. Students then complete the dispensing assessment in Week 9. Students had access to dispensing exercises and had the opportunity to seek clarification and assistance during the lectures and workshops. The study was approved by the Monash University Human Research Ethics Committee.

### 2.2. Assessment Design

In the assessment, under exam conditions, students were presented with four prescriptions assessing different classes of medication. Prescription 1 included a non-steroidal anti-inflammatory drug (NSAID) and a benzodiazepine, prescription 2 included an opioid, prescription 3 included an angiotensin-converting enzyme inhibitor (ACEi) and an eye drop and prescription 4 included an antibiotic (Abx) and hormone replacement therapy (HRT) patches. For each prescription, the class of the medications remained the same; however, variations in the specified medication as well as dosing frequency were used to maintain assessment integrity. Students who completed this assessment had access to the Australian Medicine Handbook (AMH), Australian Pharmaceutical Formulary (APF), patient dispensing records and were able to elicit further information from the virtual patient, such as concurrent medications, allergies, pregnancy and breastfeeding status and medical conditions. 

### 2.3. Data Collection

Data were extracted from the virtual dispensing program, MyDispense (MyDispense, version 5.3.32), deidentified and displayed in a Microsoft Excel^®^ spreadsheet for simplified visualization of student responses. 

In total, 3956 student first attempt responses were collected from the four MyDispense simulated prescriptions and 565 students. Prescription 1 contained 1130 responses to 12 prescription variations, prescription 2 contained 564 responses to 6 prescription variations, prescription 3 contained 1130 responses to 12 prescription variations and prescription 4 contained 1132 responses to 12 prescription variations.

### 2.4. Data Analysis

#### 2.4.1. Quantitative Data Analysis

Quantitative analysis was conducted on auto-identifiable data. Student responses for drug quantity, repeats, product, patient and prescriber selection were deemed as being correct or incorrect and not open to interpretation. Collected data were then converted to a percentage student error utilizing the formula:

% student error = (number of occurrences) ÷ (total number of attempts)

#### 2.4.2. Qualitative Data Analysis

Codebooks were developed for the analysis of the qualitative data, i.e., the student labels that required interpretation, as marking for this criterion was not automated. Through the development and use of codebooks, this study quantitatively analyzed label directions and appropriate use of ancillary labels. 

A comprehensive codebook was developed by condensing data into categories to allow patterns and trends to be more easily distinguished [14]. The use of codebooks allowed for improved efficiency, quality and reliability when analyzing the large and complex quantities of qualitative data [15].

Six codebooks were developed for the four sets of data provided. In the first phase of each codebook development, two researchers produced initial codes by reading a sample of the student responses and ideal answers for the assessed medication (familiarization). This allowed the researchers to identify criteria such as that flucloxacillin is best taken on an empty stomach. This allowed for the codebooks to reflect the criteria in the yes/no categorization. Students were required to include on the label “take on an empty stomach” OR “take 30 minutes before or 2 hours after food” AND/OR include label 3a OR label 3b (Table 3). If the student met these requirements, they would be given a “yes” code which was quantified as “1”. Conversely, if the student failed to meet these requirements, they would be given a “no” code which was quantified as “0”. For answers considered adequate but not ideal, students were given a “maybe” code which was quantified as “2”. In the second phase, the codebook was discussed with up to 3 other researchers to refine and clarify each code. In the third phase of the codebook development, another subset of data was coded independently by the two researchers, and consistency was compared to ensure rigor and robustness of the codebook. Phases two and three were repeated until consensus was reached and only “0” or “1” codes remained.

Once discrepancies were resolved, researchers then used the codebooks to analyze the four datasets. Two researchers coded each dataset to ensure rigor and robustness. Percentage of student error was calculated via the formula in Section 2.4.1.

#### 2.4.3. Harm Associated with Medication Error Classification

To further investigate how these dispensing errors could impact patient outcomes, researchers categorized the identified errors into degrees of harm. Identified errors were analyzed using the 5 references of potential harm from the Harm Associated with Medication Error Classification (HAMEC) tool (Table 1) [13].

The HAMEC tool and justification utilizing resources such as the Australian Medicines Handbook [16], MIMS Online [17], Therapeutic Guidelines [18], Pregnancy and Breastfeeding Medicines Guide [19] and the Australian Pharmaceutical Formulary [20] were used when categorizing the degree of harm to the common dispensing errors identified within the data. Factors considered during this process included the patient, medications and patient condition; patient-specific factors including age, gender, pregnancy and breastfeeding status; medication specific factors including prior use of medication, side effect profiles, therapeutic and toxicity levels; as well as medical condition factors including disease progression and severity of the disease. Two researchers categorized harm per prescription set. Variations in categorization were then discussed, and if consensus was not reached, a 3rd researcher was included to clarify and reach consensus utilizing the listed resources.

## 3. Results

### 3.1. Percentage Student Errors

Auto-identifiable errors as stated in Section 2.4.1 were not found to be frequent. In fact, errors were only found in selection of medication quantity and quantity of repeats (2% and 1% occurrence, respectively). Error margins within this domain were low and therefore not considered to be a statistically significant error. 

Analysis of student responses allowed for identification of dispensing errors within the label directions and use of ancillary labels, as well as potential harms associated with the errors in Table 2. 

#### 3.1.1. Categorization of Potential Harm 

Utilizing the HAMEC tool and professional resources, potential harm of the dispensing errors was categorized for severity (Table 2). The errors deemed to have the potential to result in “serious” or “severe” harm were errors which could put patients at risk of toxicity or inadequate treatment. Incorrect frequency was one such error identified. An example of this was 4% of students incorrectly stating the frequency of NSAIDs and HRT patches, both of which could cause “severe harm” to the patient. Additionally, if students were to omit labels 1 or 1a, it was considered to be “serious or severe harm”. Errors which were deemed to potentially cause non-life threatening, temporary harm were categorized as “minor harm”. These errors were generally seen in the route of administration, particularly when dispensing NSAIDs and eye drops. Students who made such errors when dispensing NSAIDs failed to specify “swallow whole” either on their label directions or the use of the ancillary label A (swallow whole), and for eye drops, students incorrectly used “apply”, “place” or “drop” instead of “instill” in their directions. Additionally, wrong area of application (special instruction) and removal of patch prior to applying a new patch (duration) of HRT were deemed either “minor harm” or “severe harm”. In some cases, errors have no potential to cause patient harm. One such example of this categorization is the incorrect formulation selected for NSAIDs, as the therapeutic and safety profile of tablets versus capsules are very similar, and hence deemed “no harm”.

#### 3.1.2. Route of Administration 

Label directions showed that the most common errors that occurred varied depending on the class of medication. The most common errors were the route for NSAIDs, duration and special instructions for HRT patches, with student error rates of 35%, 32.6% and 50%, respectively. The first two errors were classified as having “minor harm” as possible harms following misinterpretation were unlikely to cause significant adverse effects, whilst errors in special instructions had the potential for “minor” or “severe harm”. Overall, route of administration, frequency and formulation were among the most common dispensing errors made. 

In the analysis of the Ponstan^®^ (mefenamic acid) capsule activity, students were marked with a dispensing error if the label did not specifically state “swallow whole” in the directions or if ancillary label A (swallow whole) was not used, as this would affect the absorption of the medication. Out of 272 students who were asked to dispense Ponstan^®^ (mefenamic acid), 189 students (69%) were found to have made an error in the route of administration. On the other hand, students that wrote either “take” or “swallow whole” for Feldene^®^ (piroxicam) capsules were accepted as crushing this medication would not affect its onset and duration of action. Therefore, upon combining data from the entire cohort of 565 students, the number of students making mistakes in the route of administration was only 35%.

HRT patches had by far the widest variety among student answers, which reflected the higher rates of student error (Table 2). Of the students assessed on a HRT patch, 50% correctly identified the area of application as the lower back or abdomen (special instructions). The remaining 50% of the students were deemed to have made a dispensing error as 10% identified the incorrect site of application (e.g., affected area, upper arm, torso), 19% did not specify any site of application and 21% specified only “skin”. In total, 32% of students failed to mention “remove” or “replace” the previous patch before applying the new patch to a different area to avoid contact dermatitis (duration). 

#### 3.1.3. Drug Formulation

The incorrect formulation can have a different degree of potential harm to the patient depending on the class of medication. Of the most common errors regarding formulation, NSAIDs and antibiotics had the highest rates of student errors, with 11% and 8% of students respectively stating the wrong formulation (Table 2).

#### 3.1.4. Ancillary Labels

Ancillary labels were a component of this assessment (Table 3). Students had the option to add ancillary labels in addition to their dispensing label. Patient harm may occur when necessary labels are not utilized. Of the most common labels omitted, the majority were considered “no harm”. However, it was found that 1% of students failed to use labels 1/1a when indicated, hence categorized as potential “severe” harm as a result of lack of drowsiness warning. The severity of this potential harm is reflected in the legal requirement of this label when dispensing in Australia. The use of labels 10a on NSAIDs, 7b on eye drops and 3a/3b on antibiotics was categorized as potentially causing “minor harm” to the patient; 4%, 14% and 5% of students respectively did not include these labels (Table 2).

## 4. Discussion

This retrospective study demonstrated that the most common dispensing errors in first-year pharmacy students occurred largely in the label directions where errors were found in the route of administration, formulation and frequency of dosing. The percentage of students who made an error within these three domains was similar to those reported in the literature. Both Aldhwaihi et al. [21] and James et al. [4] conducted paper and article reviews, describing some of the most frequent errors within previous studies to be drug formulation and frequency. 

Aldhwaihi et al. [21] and James et al. [4] also found that incorrect medication selection, strength and quantity were among some of the commonly reported errors in both hospital and community pharmacy. This was further supported by Darbishire et al. [5], who found incorrect patient selection to also be a common error. Conversely, in our study, there was either a 0% incidence rate or an error rate so small it was deemed insignificant for these error types. It should be noted that the aforementioned studies were conducted in “real-life” setting with practicing pharmacists, while our study was based a virtual dispensing simulator and pharmacy students. The discrepancy is likely due to the dispensing event being carried out as an assessment; as such, students may have focused more on aspects of dispensing they were aware they were being assessed on. Additionally, given that the assessment was carried out on dispensing simulator, patient/prescriber profiles and formularies may not have been as populated as a “live” dispensing environment, resulting in fewer opportunities for selection errors. It should also be noted that the students completed the assessment in a controlled environment, free of distractions common in pharmacy; therefore, comparing this to that of the working environment within a community or hospital pharmacy setting can be difficult. Previous research highlighted several external factors which may have been associated with a higher incidence of dispensing errors. These disruptions, namely high workload, inadequate staffing, pharmacists being interrupted and excessive noise [22,23], are limited when using a virtual pharmacy software, thus reducing the incidence for these selection errors in students.

Furthermore, our results identified errors in the use of ancillary labels and special instructions despite students dispensing medications in a more controlled environment. These errors can possibly be explained by the lack of experience in first-year students. These students have not yet been exposed to any therapeutic courses or been on experiential placements and as such may be unfamiliar with possible side effects, interactions or special instructions common to the assessed medication classes. They were relying mostly on the information provided on the prescription when generating the dispensing label. While the APF lists recommended ancillary labels, students unfamiliar with the reference text would find it challenging to navigate to the information required. Inappropriate use of ancillary labels was an aspect of the dispensing process that had very limited literature given the variability in requirements across different countries and the fact that the studies that were previously reviewed did not address this type of error. 

It should be noted that dispensing errors of the highest frequencies, such as NSAID route (35%), HRT duration (32.9%) and HRT special instructions (50%), while high, were all categorized to be “minor harm”, with the exception of the 40% who did not clearly specify site of HRT application, which could lead to potentially grave consequences if applied to the breasts. This was therefore categorized as “severe” harm. In comparison, errors considered to have the potential to cause “severe harm” generally occurred at a much lower frequency, consistent with the Heinrich proposal that incidences of no harm or minor harm occur at a much greater frequency than serious events [24]. This was also noted in the systematic review by Aldhwaihi et al. [21]. The highest frequency for a severe dispensing error was 10.4% for the frequency of benzodiazepine dosing. This indicates that students recognize the severity of potential errors and attempt to minimize the occurrence of these errors. 

A limitation of this study was that the study participants included first-year pharmacy students only. First-year students have limited therapeutic knowledge and experience compared to practicing pharmacists when it comes to dispensing and may not have an adequate understanding of medications. For instance, there was a number of students who made vague and/or incorrect directions for the area of application for HRT patches, while some students included both labels 3a and 3b (“Take on an empty stomach at least half an hour before meals and at bedtime” OR “Take on an empty stomach at least half an hour before food or two hours after food”) for flucloxacillin. As such, this caused a high percentage of students to make errors in optimal ancillary label use and providing clear label directions. These are errors that would be less common with more extensive knowledge and experience, making it difficult to generalize the findings to those already working as healthcare professionals. While the codebook developed in this study allowed for quantitative analysis of student responses and the HAMEC tool allowed for structured categorization of harms, validation of these tools would be warranted.

Dispensing errors are a common occurrence within the pharmacy profession, but avoidable. Early identification of the types of errors and possible contributing factors will help to guide potential strategies to help to reduce these incidences. Students have not had the same repetitive engagement in the dispensing process as practicing pharmacists; as such, more exposure, practice and support would help to minimize these errors. Furthermore, through the results of this study, considerations of possible strategies or curriculum changes can be made. For instance, a more comprehensive tutorial on how to interpret the dispensing guides such as the APF would allow students to confidently select appropriate ancillary labels. Additionally, a focus into the definitions of Latin medical abbreviations such as “qid”, “tds” or “bd” which are often seen on prescriptions would help to reduce errors in relation to the frequency of dosing on the label. Exposure to resources on counseling information from sources such as medication formularies (e.g., the Australian Medicine Handbook) or in Consumer Medicine Information leaflets would provide students with specific medication knowledge such as HRT patch application sites. As such, the results of this study may be used to improve safe dispensing skills in first-year pharmacy students or to assess the longitudinal dispensing skill development of students.

## Figures and Tables

**Table 1:** The Harm Associated with Medication Errors Classification (HAMEC) tool—potential harm [13].

Stratification of Harm	Degree of Harm	Description
0	None	No potential for patient harm, nor any change in patient monitoring, level or length of care required
1	Minor	Potential for minor, non-life threatening, temporary harm that may or may not require patient monitoring. There may or may not be minimal increase in length of care (<1 day)
2	Moderate	Potential for minor, non-life threatening, temporary harm that requires patient monitoring. There may or may not be minimal increase in length of care (<1 day)
3	Serious	Potential for major, non-life threatening, temporary harm or minor permanent harm that would require a high level of care such as the administration of an antidote. An increase in the length of care of ≥1 day is expected.
4	Severe	Potential for life-threatening or mortal harm or major permanent harm that would require a high level of care such as the administration of an antidote or transfer to intensive care. A substantial increase in the length of care of >1 day is expected.

**Table 2:** Frequency and potential harm associated with dispensing errors.

	Medication Class % of students, (Harm)						
	NSAID	BZ	Opioid	Eye Drops	ACEi	Abx	HRT
Label Directions							
Route	35%, (1)	0.2%, (1)	2.5%, (1)	5.5%, (1)	0.5%, (1)	0.5%, (1)	3.0%, (1)
Quantity	1.1%, (4)	0.2%, (4)	0.5%, (4)	0.5%, (4)	0.2%, (4)	0%, (4)	1.2%, (4)
Frequency	4.4%, (4)	10.4%, (4)	1.6%, (4)	1.4%, (4)	0%, (4)	1.2%, (4)	4.4%, (4)
Formulation	11.3%, (0)	1.9%, (0)	0.7%, (1)		0.2%, (1)	7.8%, (0)	1.4%, (1)
Duration	1.7%, (1)					0.4%, (3)	32.6%, (1)
Special Instructions	1.8%, (1)	11%, (0)		0.7%, (4)		2.9%, (1)	10, 19, 21%, (1, 4, 4)
Ancillary labels *	6.4, 4.4%, (0, 1)	0.7%, (4)	3.2, 1.4%, (1, 4)	14.3, 14.1%, (0, 1)	9.8, 3.4, 0.4%, (0, 1, 3)	5.0%, (1)	22.0%, (0)

* = may include more than 1 ancillary label. NSAID = non-steroidal anti-inflammatory, BZ = benzodiazepine, ACEi = angiotensin converting enzyme inhibitor, Abx = antibiotics, HRT = hormone replacement therapy. 0 = no harm, 1 = minor harm, 2 = moderate, 3 = serious, 4 = severe.

**Table 3:** Ancillary (Cautionary Advisory Labels) used in the MyDispense assessment [20].

Label	Cautionary Advice
A	SWALLOW WHOLE Do not crush or chew
K	FOR EXTERNAL USE ONLY
1/1a	This medicine may cause DROWSINESS and may increase the effects of alcohol. If affected, do not drive a motor vehicle or operate machinery. This preparation is to aid sleep. Drowsiness may continue the following day. If affected, do not drive or operate machinery. Avoid alcohol.
3a/3b	Take on an empty stomach at least half an hour before meals and at bedtime Take on an empty stomach at least half an hour before food or two hours after food
7b	Discard… days after opening. Date opened / /
10a	Do not take more than one aspirin tablet or capsule each day while being treated with this medicine.
11	DO NOT TAKE POTASSIUM while being treated with this medicine unless advised by your doctor
12	This medicine may affect mental alertness and/or coordination. If affected, do not drive a motor vehicle or operate machinery.
16	This medicine may cause dizziness especially when you stand up quickly. Ask your doctor or pharmacist for advice.
18	Avoid eating grapefruit or drinking grapefruit juice while being treated with this medicine.

## Data Availability

The data are available from the authors upon reasonable request.
